# Supplementary material for: Rapid turnover of life-cycle-related genes in the brown algae
Source: Genome Biol. 2019 Feb 14;20:35. doi: 10.1186/s13059-019-1630-6 (PMC6374913; doi:10.1186/s13059-019-1630-6)
Supplement: Supplementary file 3 — Protein alignments, corresponding Gblocks html file and CDS sequences for the lineage conserved gametophyte-biased genes. (ZIP 2109 kb) [file 13059_2019_1630_MOESM3_ESM.zip › Con_GA_alignments/Ec-19_002630.aa_ali.fasta-gb.htm]

Ec-19\_002630.aa.fasta.trans.aa\_ali.fasta


## Gblocks 0.91b Results

Processed file: **Ec-19\_002630.aa.fasta.trans.aa\_ali.fasta**  
Number of sequences: **2**  
Alignment assumed to be: **Protein**  
New number of positions: **446** (selected positions are underlined in blue)

```
                         10        20        30        40        50        60
                 =========+=========+=========+=========+=========+=========+
Ec-19_002630     MSPAAETGSHIFMPLPQAGSGLRIAAQPQWAPGHAPAVAMTAVGIPPGSGAALSERDRRL
MSTRG.30635.1    ------------------------------------------------------------
                                                                             


                         70        80        90       100       110       120
                 =========+=========+=========+=========+=========+=========+
Ec-19_002630     AARHEERRRRSLHGPQEGPADKVFQEREQERQAFEREAAASNRSVNAERAGSTRNEGGHP
MSTRG.30635.1    ------------------------------------------------------------
                                                                             


                        130       140       150       160       170       180
                 =========+=========+=========+=========+=========+=========+
Ec-19_002630     RSHGHETSSTVRAVVPIPAAGGASLTSALDQKVADKSSREAKYEEKRQQYLAAQRGSTSG
MSTRG.30635.1    ------------------------------------------------------------
                                                                             


                        190       200       210       220       230       240
                 =========+=========+=========+=========+=========+=========+
Ec-19_002630     EPRVRFQGNQAQESSVMSGHSPALVAPTIREVQQSASDQQQNVDPTELPNAPSPGAKIQR
MSTRG.30635.1    --------------------------------------------------VPRSRPRAQG
                                                                             


                        250       260       270       280       290       300
                 =========+=========+=========+=========+=========+=========+
Ec-19_002630     KPGIDETSGRVEQARFPPQEAKRAPSSGPLRLPEQGNLPRRQPNPTMAGVARPDSDYARH
MSTRG.30635.1    IL---------------------QLEEGPVPLPKD------NEN----------PHFPKR
                                                                             


                        310       320       330       340       350       360
                 =========+=========+=========+=========+=========+=========+
Ec-19_002630     VREQMMAGVAAAQRGNDDARRPGPVAATIPADRGRTLPHAQPVETEDGTTTVSSTIDRKA
MSTRG.30635.1    DREQMVSDAMIVQLAKDNEHRSAHDVAPTPAEQGSGFMHTSAVGKGGTESSVPASVDRKA
                  ###########################################################


                        370       380       390       400       410       420
                 =========+=========+=========+=========+=========+=========+
Ec-19_002630     EYASQLREQMASDEATRKAMEIERKCKVSTVSSAAGPAIGEHEEGLRGGEDEWV-NESVR
MSTRG.30635.1    EYACQLREQMATDEATKKAAENERKNLSTITSSTGATRGGVDGSGARGDGSEWMVGGPGR
                 #####################################################      #


                        430       440       450       460       470       480
                 =========+=========+=========+=========+=========+=========+
Ec-19_002630     QAKAEYAQQLREQMAAKENAQRAAKGEMENSRSANAGPSWIEGATEGRKARRRRSNTEYA
MSTRG.30635.1    DAKAEYAQQLREQMAAKENERRAAKEKMQRSLSANAGPRWIEGATEGREARRKRSNTEYA
                 ############################################################


                        490       500       510       520       530       540
                 =========+=========+=========+=========+=========+=========+
Ec-19_002630     EQLRAQIAAQKSINQAQQSLVAARVQPPKDMFEQGGYEKYY--QQLHQRLLGGVSS--FD
MSTRG.30635.1    EQLRAQIAAQKDLTRAQQSRIASRAQAPRPSFLDAGHNDEYQQQQLHHQPQWGVDVGHRN
                 #########################################  ###########      


                        550       560       570       580       590       600
                 =========+=========+=========+=========+=========+=========+
Ec-19_002630     YQSGEPERRRSQIDD-HRSQ---------GRPRTEEKISLSRGEVSNPLATARQERVNHQ
MSTRG.30635.1    LRNTGPERLQAGHDQGHGGQDRREKEHSEGLVDAGWKVPASKRGADNTLALERQKRLDNQ
                                              ###############################


                        610       620       630       640       650       660
                 =========+=========+=========+=========+=========+=========+
Ec-19_002630     REYAEALRRDMAAPKVDSLGRPIGDITSPSRTRLRNSNSGTRNTNNNPEFFGQNYGNSAP
MSTRG.30635.1    RDYAEALRRDMATPKVDSLGRPIEDITSPSRTRFRNAHNQSRYPTTDGVLSRGGGEIGVT
                 ##########################################                  


                        670       680       690       700       710       720
                 =========+=========+=========+=========+=========+=========+
Ec-19_002630     ----REAGHLQPGPRPQAGEHVRDWSQQPFE-REYLREHATPYGAWPSSQAQSQGPYEVG
MSTRG.30635.1    GEGRGSLSHQRYHVDGDMGQQIDRGRRPPAQGGEYRGDQRSQTAAWPPSQDR---AWYAG
                                                                             


                        730       740       750       760       770       780
                 =========+=========+=========+=========+=========+=========+
Ec-19_002630     EHRHSRQ-PPVPGTHYEAYGSNPKGYPSRGDYDELEYPTGRRGNAKWVADGISASGLPPP
MSTRG.30635.1    EQPQQRQWLPFPETHRMTHGEGANEHHSGSGYERRRPENGQRG-VRWVGDVGGVHGLPPP
                          ##################################   ##############


                        790       800       810       820       830       840
                 =========+=========+=========+=========+=========+=========+
Ec-19_002630     RRDFVDDGERRGERWGTSGP------YPERGGGYPGNRTRLPEFGGKSWDITNPSVG---
MSTRG.30635.1    QGPYANGG--REQSWGTSGPSTLGGGYPDRASGCWSYPGQQQDDGAQNLLARGASDGTWR
                 ########  ##########                                        


                        850       860       870       880       890       900
                 =========+=========+=========+=========+=========+=========+
Ec-19_002630     --GGHDR--NVHPGPIRDHREQQDRRYFGPRGPPGGAPAAGGGVGVRSTKASTMHNPVDP
MSTRG.30635.1    GEGGWNGNGGIHPGPPWEARGQQQYRHSGPGGTPGGAPAV--GVAVVSTKASTMHNPVDP
                            ############################   ##################


                        910       920       930       940       950       960
                 =========+=========+=========+=========+=========+=========+
Ec-19_002630     EELARIQAKKESYRRDLEAQMAEHTKLQDQRKQGVPGSKEAEVFHLQKAMEERSERNKMA
MSTRG.30635.1    DELARIQAKKESYRRDLEAQMAAHKRILDQRKKGAP------------------------
                 ####################################                        


                        970       980       990      1000      1010      1020
                 =========+=========+=========+=========+=========+=========+
Ec-19_002630     PGYEIGPLGVPVIKDNYSTTGRGQRSPAKMRAVAPAATGNQGLSASSAIDPSSGPVGEST
MSTRG.30635.1    ------------------------------------------------------------
                                                                             


                       1030      1040      1050      1060      1070      1080
                 =========+=========+=========+=========+=========+=========+
Ec-19_002630     PQTSSELFGSGHDGTSSSLLAPSGGGADVSDAEGVGAPSRRGSGLRELLGIETNAERDRE
MSTRG.30635.1    ------------------------------------------------------------
                                                                             


                       1090      1100      1110      1120      1130      1140
                 =========+=========+=========+=========+=========+=========+
Ec-19_002630     KSRVQAEALKKQVEDNKVRRDIQKAQLELHERKEELRLQKEREQLHERYEQEGKLAKALE
MSTRG.30635.1    ------------------------------------------------------------
                                                                             


                       1150      1160      1170      1180      1190      1200
                 =========+=========+=========+=========+=========+=========+
Ec-19_002630     AEKLLKEAEEARLAHAARKEKAAAESREEEKREDARVEREQKELERKYARERAAEEPLVQ
MSTRG.30635.1    ------------------------------------------------------------
                                                                             


                       1210      1220      1230      1240      1250      1260
                 =========+=========+=========+=========+=========+=========+
Ec-19_002630     ASSNPANLPVSEFPTKKGGGTRSPRPIKSPRELEVNEKRHEDSVMMPHLPGRERGTPSIE
MSTRG.30635.1    ------------------------------------------------------------
                                                                             


                       1270      1280      1290      1300      1310      1320
                 =========+=========+=========+=========+=========+=========+
Ec-19_002630     AQSDNGEQMVEPSKIETKGPVQSDGPSPHQPPASPRGLATPREGVSIEKLRTELWKQKQL
MSTRG.30635.1    ------------------------------------------------------------
                                                                             


                       1330      1340      1350      1360      1370      1380
                 =========+=========+=========+=========+=========+=========+
Ec-19_002630     TEVLRHEVGQQEAEKRKLIDIARVAKESEAAALRAAYERRASKHPPAKELGIAGGGAEGN
MSTRG.30635.1    ------------------------------------------------------------
                                                                             


                       1390      1400      1410      1420      1430      1440
                 =========+=========+=========+=========+=========+=========+
Ec-19_002630     ELWRVPPPPSTEGAEGEGRTPSPSARRRAESNFAAKNAYVANRKFDTDETAEDRLLLAGL
MSTRG.30635.1    ------------------------------------------------------------
                                                                             


                       1450      1460      1470      1480      1490      1500
                 =========+=========+=========+=========+=========+=========+
Ec-19_002630     RRLSDGNRAPGGGCDTDRLRKDDLGGDSTFREQVPGARAWSSGAVSNSRDGRDSKRRPLD
MSTRG.30635.1    ------------------------------------------------------------
                                                                             


                       1510      1520      1530      1540      1550      1560
                 =========+=========+=========+=========+=========+=========+
Ec-19_002630     YGAVSPGGIRAESRYVFPDGSVRRRLETPAAEGGVGDGGGGGGGVPMPLVGTSRIASADL
MSTRG.30635.1    ------------------------------------------------------------
                                                                             


                       1570      1580      1590      1600      1610      1620
                 =========+=========+=========+=========+=========+=========+
Ec-19_002630     SFALGIDSLRGGGNGAAGDDVEKASWRRRGTGRTGSRDNEGGEHRGPGAAPGAEWAPVGR
MSTRG.30635.1    ------------------------------------------------------------
                                                                             


                       1630      1640      1650      1660      1670      1680
                 =========+=========+=========+=========+=========+=========+
Ec-19_002630     PPRAFVPFLNLPVSNGKANGNNSKEGRWASRDSDGSDSPYFLPPVYSAAVNGRSKRRGRP
MSTRG.30635.1    ------------------------------------------------------------
                                                                             


                       1690      1700      1710      1720      1730      1740
                 =========+=========+=========+=========+=========+=========+
Ec-19_002630     LAGIGGVLVSPGRAMLTGPGRPGTGATSPERPAYGETGSGHHRRRQSRQRARREGADLGS
MSTRG.30635.1    ------------------------------------------------------------
                                                                             


                       1750      1760      1770      1780      1790      1800
                 =========+=========+=========+=========+=========+=========+
Ec-19_002630     TSIGDEEFSDDCESVGSGSTDVEFLRRRAEAKLKSLDRREAAEKAKEKELNLLVASSPAR
MSTRG.30635.1    ------------------------------------------------------------
                                                                             


                       1810      1820      1830      1840      1850      1860
                 =========+=========+=========+=========+=========+=========+
Ec-19_002630     GTTDVTTPEAPAMHSRAVGGVRLALPTHNTARRTISAASSSSSRSLMYSRRKKRPGTNKS
MSTRG.30635.1    ------------------------------------------------------------
                                                                             


                       1870
                 =========+=====
Ec-19_002630     ESSMLGDSRWAEQLK
MSTRG.30635.1    ---------------
```

```
Parameters used
Minimum Number Of Sequences For A Conserved Position: 2
Minimum Number Of Sequences For A Flanking Position: 2
Maximum Number Of Contiguous Nonconserved Positions: 8
Minimum Length Of A Block: 10
Allowed Gap Positions: None
Use Similarity Matrices: Yes
```

```
Flank positions of the 9 selected block(s)
Flanks: [302  413]  [420  521]  [524  534]  [570  642]  [730  763]  [767  788]  [791  800]  [852  879]  [883  936]  

New number of positions in Ec-19_002630.aa.fasta.trans.aa_ali.fasta-gb:  446  (23% of the original 1875 positions)
```
